# Unique properties of thymic antigen-presenting cells promote epigenetic imprinting of alloantigen-specific regulatory T cells

**DOI:** 10.18632/oncotarget.16221

**Published:** 2017-03-15

**Authors:** Garima Garg, Eirini Nikolouli, Matthias Hardtke-Wolenski, Aras Toker, Naganari Ohkura, Michael Beckstette, Takahisa Miyao, Robert Geffers, Stefan Floess, Norbert Gerdes, Esther Lutgens, Anke Osterloh, Shohei Hori, Shimon Sakaguchi, Elmar Jaeckel, Jochen Huehn

**Affiliations:** ^1^ Department of Experimental Immunology, Helmholtz Centre for Infection Research, Braunschweig, Germany; ^2^ Department of Gastroenterology, Hepatology, Endocrinology, Hannover Medical School, Hannover, Germany; ^3^ Department of Experimental Immunology, World Premier International Immunology Frontier Research Center, Osaka University, Suita, Japan; ^4^ Department of Experimental Pathology, Institute for Frontier Medical Sciences, Kyoto University, Kyoto, Japan; ^5^ Laboratory for Immune Homeostasis, RIKEN Center for Integrative Medical Sciences, Yokohama City, Kanagawa, Japan; ^6^ Genome Analytics, Helmholtz Centre for Infection Research, Braunschweig, Germany; ^7^ Institute for Cardiovascular Prevention, Ludwig-Maximilians-University, Munich, Germany; ^8^ Division of Cardiology, Pulmonology, and Vascular Medicine Medical Faculty, University Hospital Düsseldorf, Düsseldorf, Germany; ^9^ Department of Medical Biochemistry, Academic Medical Center, University of Amsterdam, AZ, Amsterdam, The Netherlands; ^10^ Department of Immunology, Bernhard Nocht Institute for Tropical Medicine, Hamburg, Germany

**Keywords:** regulatory T cells, thymic APCs, epigenetic modification, alloantigen-specificity, Immunology and Microbiology Section, Immune response, Immunity

## Abstract

Regulatory T cells (Tregs) are potential immunotherapeutic candidates to induce transplantation tolerance. However, stability of Tregs still remains contentious and may potentially restrict their clinical use. Recent work suggested that epigenetic imprinting of *Foxp3* and other Treg-specific signature genes is crucial for stabilization of immunosuppressive properties of Foxp3^+^ Tregs, and that these events are initiated already during early stages of thymic Treg development. However, the mechanisms governing this process remain largely unknown. Here we demonstrate that thymic antigen-presenting cells (APCs), including thymic dendritic cells (t-DCs) and medullary thymic epithelial cells (mTECs), can induce a more pronounced demethylation of *Foxp3* and other Treg-specific epigenetic signature genes in developing Tregs when compared to splenic DCs (sp-DCs). Transcriptomic profiling of APCs revealed differential expression of secreted factors and costimulatory molecules, however neither addition of conditioned media nor interference with costimulatory signals affected Foxp3 induction by thymic APCs *in vitro*. Importantly, when tested *in vivo* both mTEC- and t-DC-generated alloantigen-specific Tregs displayed significantly higher efficacy in prolonging skin allograft acceptance when compared to Tregs generated by sp-DCs. Our results draw attention to unique properties of thymic APCs in initiating commitment towards stable and functional Tregs, a finding that could be highly beneficial in clinical immunotherapy.

## INTRODUCTION

CD4^+^ regulatory T cells (Tregs) - characterized by the expression of the lineage specification factor Foxp3 - play an indispensable role for the maintenance of immune homeostasis and self-tolerance [1]. In addition, Foxp3^+^ Tregs also represent a cell type that is highly relevant for clinical considerations, e.g. for the treatment of autoimmune conditions, graft-versus-host disease (GvHD) and allograft rejection [2–7], and the efficiency of Tregs in decreasing the incidence of acute GvHD in first clinical trials have encouraged further use of these cells in cellular therapies [8]. Foxp3^+^ Tregs can be generated in large numbers *in vitro* by stimulating conventional CD4^+^ T cells in presence of TGF-β (*in vitro* induced Tregs, iTregs) [9], but stability of Foxp3 expression and suppressive potential of these cells after adoptive transfer is discussed controversially [10–13]. Thus, generation of Tregs with a stable immunosuppressive phenotype is crucial to render their use in therapeutic approaches feasible [14]. This is especially true in case of adoptive Treg therapy for autoimmune diseases and after transplantation which will likely require the use of antigen-specific Tregs that could turn into detrimental effector cells if the regulatory phenotype is lost.

Stability of Foxp3 expression correlates with DNA demethylation at a conserved intronic CpG-rich region within the Foxp3 locus, designated Treg-specific demethylated region (TSDR) [15]. Demethylation at the TSDR (also known as CNS2) is not required for initiation of Foxp3 expression, but for its long-term maintenance [10, 16, 17]. However, stable Foxp3 expression is not sufficient for fully functional Tregs. Instead, selective demethylation of a number of Treg-specific signature genes including *Ctla4*, *Gitr*, *Eos* and *Helios* in addition to the TSDR is crucially required for Foxp3^+^ T cells to acquire Treg-specific gene expression, lineage stability and full suppressive activity [13, 18, 19]. Accordingly, iTregs with fully methylated Treg-specific epigenetic signature genes display only instable Foxp3 expression and lack suppressive capacity upon adoptive transfer *in vivo* [10, 11, 13]. Hence, understanding those mechanisms that cause selective demethylation of Treg-specific epigenetic signature genes in developing Tregs could open up ways to manipulate the DNA methylation status of Tregs and allow safe application of *in vitro* generated Tregs for therapeutic approaches [20].

Although it is known that selective demethylation of the TSDR and other Treg-specific epigenetic signature genes is initiated already during early stages of thymic Treg development [13, 21, 22], cellular players and molecular mechanisms governing epigenetic imprinting of the Treg fate within the thymus remain largely enigmatic. It is tempting to speculate that thymic antigen-presenting cells (APCs) are involved in this process since thymic Treg development requires CD4SP (CD4 single positive) thymocytes encountering their cognate antigen presented by thymic APCs together with proper costimulation and cytokine signaling [23–36].

To investigate the role of thymic APCs, we focused on medullary thymic epithelial cells (mTECs) and thymic dendritic cells (t-DCs), those APCs that are predominantly found in the thymic medulla, the main hub of Foxp3^+^ Treg development [37]. We demonstrate that both mTECs and t-DCs not only have a preferential ability to induce alloantigen-specific Foxp3^+^ Tregs *in vitro* (allo-iTregs) when compared to splenic DCs (sp-DCs), but also to initiate a more pronounced demethylation of the TSDR and other Treg-specific epigenetic signature genes. Transcriptomic profiling of APCs uncovered differential expression of numerous immunologically relevant molecules, however neither secreted factors nor major costimulatory signals seemed to be functionally relevant for induction of Foxp3^+^ Tregs in *in vitro* allogeneic cultures. Importantly, thymic APC-induced allo-iTregs showed a superior suppressive capacity when tested in a highly immunogenic, allogeneic skin transplantation model. Viewed as a whole, our results demonstrate that thymic APCs harbor unique properties, which are instrumental for the epigenetic imprinting and stabilization of Foxp3^+^ Tregs.

## RESULTS

### Thymic APC-induced Foxp3^+^ Tregs show highest demethylation at Treg-specific epigenetic signature genes

**Figure 1:**
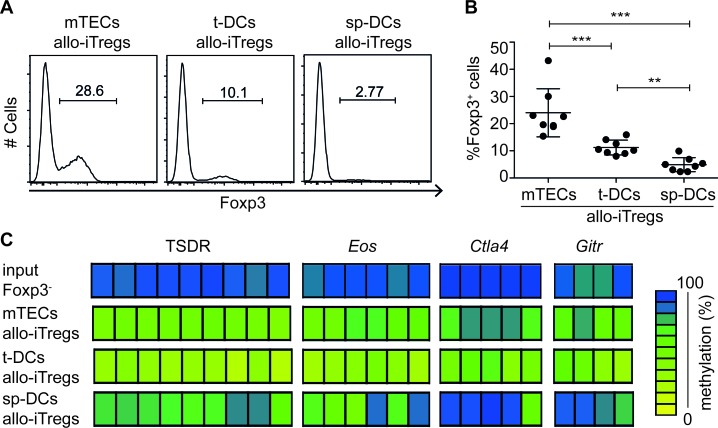
Thymic APCs very efficiently induce allo-iTregs with highest demethylation at TSDR and other Treg-specific epigenetic signature genes Indicated APCs isolated from BALB/c mice were cultured with allogeneic CD4SP Foxp3^RFP−^ thymocytes isolated from *Foxp3^RFP^ x IL-10^GFP^* double reporter mice (C57BL/6 background) in presence of IL-2 for six days. **A.** Expression of Foxp3 in cultured CD4^+^CD90^+^ thymocytes was analyzed by flow cytometry. Numbers indicate frequency of Foxp3^+^ cells. Representative data from one out of eight independent experiments are shown. **B.** Graph shows frequency of Foxp3^+^ allo-iTregs from cultures with indicated APCs. Data are summarized from eight independent experiments (mean ± SD) and tested for significance using Mann-Whitney test; **p < 0.01; ***p < 0.001. **C.** At day 6, Foxp3^RFP+^ allo-iTregs were sorted from indicated cultures, and genomic DNA isolated from these cells was analyzed for the methylation status of TSDR, *Eos*, *Ctla4* and *Gitr*. Representative data from one out of six (TSDR), two (*Gitr*) or three (*Eos* and *Ctla4*) independent experiments are depicted. Genomic DNA from CD4SP Foxp3^−^ thymocytes was analyzed as input control for the methylation status of TSDR (three independent samples) and *Eos*, *Ctla4* and *Gitr* (unicate). Each bar represents one CpG motif. The degree of methylation at each CpG motif is represented according to the color code.

Previous studies have demonstrated that selective demethylation of the TSDR and other Treg-specific epigenetic signature genes is initiated already during early stages of thymic Treg development [13, 21, 22]. To investigate the specific contribution of thymic APCs to this process, an alloantigen-specific *in vitro* stimulation system was established. As stimulators, we chose APCs from the medulla since this region is the major site of thymic Foxp3^+^ Treg development [37]. CD45^−^EpCAM^+^Ly51^−^ mTECs and CD45^+^CD11c^hi^Lin^−^ DCs (t-DCs) were isolated *ex vivo* from thymi of BALB/c mice, and CD45^+^CD11c^hi^Lin^−^ DCs from spleens (sp-DCs) of BALB/c mice were taken as controls (sp-DCs) (Supplementary Figure S1A). As responder cells, CD4SP Foxp3^−^ thymocytes were purified from Foxp3 reporter mice (C57BL/6 background) and cultured with mTECs, t-DCs or sp-DCs for six days in presence of exogenous IL-2, resulting in a substantial and comparable proliferation of responder cells in all cultures (data not shown). First, we studied the capacity of the different APCs to induce Foxp3 expression in the responder cells. Flow cytometric analysis revealed that both mTECs and t-DCs had induced significantly higher frequencies of Foxp3^+^ allo-iTregs as compared to sp-DCs (Figure 1A, 1B). Importantly, the same optimal ratio of APCs to thymocytes was used for t-DCs and sp-DCs (1:10), while mTECs induced maximal percentages of allo-iTregs at a ratio of 1:50 (Supplementary Figure S1B). Hence, for all further allo-iTreg induction experiments these optimal ratios were used. As expected from a previous report [38], peripheral CD4^+^Foxp3^−^ T cells isolated from spleen and lymph nodes failed to efficiently differentiate into allo-iTregs upon coculture with thymic APCs (Supplementary Figure S1C). However, when CD4SP Foxp3^−^ thymocytes, containing CD25^+^Foxp3^−^ Treg precursors [23–36], were cultured with syngeneic APCs for six days in presence of exogenous IL-2 as described previously [28], we also observed that both mTECs and t-DCs had induced higher frequencies of Foxp3^+^ Tregs as compared to sp-DCs (Supplementary Figure S1D). Thus, CD4SP Foxp3^−^ thymocytes displaying an intrinsically high ability to develop into Foxp3^+^ Tregs were chosen for all future experiments.

Having shown that thymic APCs have a higher capacity to induce Foxp3^+^ allo-iTregs as compared to sp-DCs, we next asked for the methylation status of the Treg-specific epigenetic signature genes in these Foxp3^+^ allo-iTregs. Genomic DNA was isolated from sort-purified Foxp3^+^ allo-iTregs, bisulfite treated and analyzed by pyrosequencing. Notably t-DC-induced, but also mTEC-induced allo-iTregs showed a pronounced demethylation of the TSDR and other Treg-specific epigenetic signature genes including *Eos*, *Ctla4* and *Gitr*, whereas CD4SP Foxp3^−^ thymocytes analyzed as input controls were fully methylated at all epigenetic signature genes (Figure 1C and Supplementary Figure S1E) as expected from previous studies [13, 21]. Interestingly, sp-DC-induced allo-iTregs showed a weaker demethylation at TSDR, *Eos*, *Ctla4* and *Gitr*, suggesting that only thymic APCs can initiate a pronounced demethylation of Treg-specific epigenetic signature genes in allo-iTregs, with t-DCs being the most efficient APC. Together, these data imply that thymic APCs not only have a preferential ability to induce Foxp3 expression, but also to provoke the epigenetic remodeling during thymic Treg development.

### Transcriptional profiling of mTECs, t-DCs and sp-DCs identifies various differentially expressed genes

**Figure 2:**
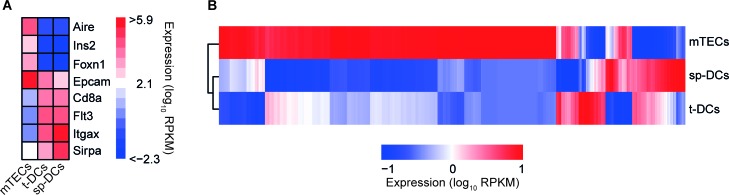
Global view on transcriptomes of thymic APCs *Ex vivo* isolated mTECs, t-DCs and sp-DCs (all from BALB/c mice) were transcriptionally profiled by RNA-Seq. **A.** Heatmap analysis for expression of cell-type specific signature genes for log_10_ transformed data. Bars are color-coded according to the expression value RPKM as indicated in expression scale. Each bar is an average of three independent experiments. **B.** Hierarchical clustering of mean averaged over replicates and scaled gene expression values (RPKM) of all profiled genes with *DESeq2* estimated |log_2_FC| of at least 1.5 and adjusted *p*-value of at most 0.05 in at least one of the performed comparisons. Bars are color-coded according to the expression value RPKM as indicated in the expression scale. Each bar is an average of three independent experiments.

To unravel the unique features of thymic APCs in driving differentiation of a stable Treg lineage, we performed a transcriptional profiling by RNA-Seq. To this end, mTECs and t-DCs were isolated *ex vivo* to high purity and sp-DCs were taken as controls (Supplementary Figure S1A). To validate the specificity of the RNA-Seq analysis, a set of previously defined control genes for mTECs and DCs was analyzed in detail. As expected, mTECs showed a higher expression of *Aire*, *Ins2*, *Foxn1* and *Epcam* when compared to both t-DCs and sp-DCs, which in turn showed a higher expression of *Cd8a*, *Flt3*, *Itgax* and *Sirpa* (Figure 2A). Global inspection of the RNA-Seq data by principal component analysis (PCA) and clustering of Euclidian sample distances revealed homogeneous and clearly separated clusters of sample replicates and showed a higher similarity between the two DC populations as compared to mTECs (Supplementary Figure S2). Hierarchical clustering of all differentially expressed genes (DEGs) amongst the analyzed APCs corroborated this finding (Figure 2B). Accordingly, the largest number of DEGs was found in the comparisons of mTECs with t-DCs (1555 up- and 8800 down-regulated in t-DCs) or mTECs with sp-DCs (1719 up- and 9046 down-regulated in sp-DCs) (Supplementary Table S1). Although the number of DEGs was substantially smaller when comparing sp-DCs with t-DCs (3131 up- and 1181 down-regulated in t-DCs), yet a high degree of differential gene expression could be observed. The results from these pairwise comparisons were fuelled into a KEGG pathway and GO enrichment analysis to identify functional pathways that contribute to the unique properties of thymic APCs in fostering the generation of stable Foxp3^+^ Tregs. Among the numerous overrepresented KEGG pathways and GO categories we focussed on those involved in cellular communication like cytokine-cytokine receptor interaction (KEGG ID: 4060), cell adhesion (KEGG ID: 4514; GO ID: 5925, 7155) and plasma membrane location (GO ID: 5886, 5887, 7166, 9897, 9986, 16020, 16021, 16324) (Supplementary Tables S2, S3). From these pathways and categories, immunologically relevant molecules were extracted and grouped into either cytokines/cytokine receptors and chemokines/chemokine receptors (Supplementary Figure S3) or cell surface/cell adhesion-associated molecules and costimulatory molecules (Supplementary Figure S4). Detailed inspection of these extracted RNA-Seq data revealed that many genes were expressed at similar levels in both t-DCs and sp-DCs (e.g. *Il1b*, *Il21r*, *Il10ra*, *Xcr1*), confirming the more pronounced transcriptional variation between mTECs and DCs. However, t-DCs and sp-DCs still presented differential expression of a substantial number of immunologically relevant genes, and some genes, particularly those belonging to the group of costimulatory molecules (Supplementary Figure S4B), even displayed higher expression levels in both thymic APC subsets as compared to sp-DCs, making them interesting candidates contributing to the unique features of thymic APCs in driving differentiation of a stable Treg lineage.

### Neither costimulatory signals nor secreted factors contribute to efficient induction of allogeneic Foxp3^+^ Tregs by thymic APCs

**Figure 3:**
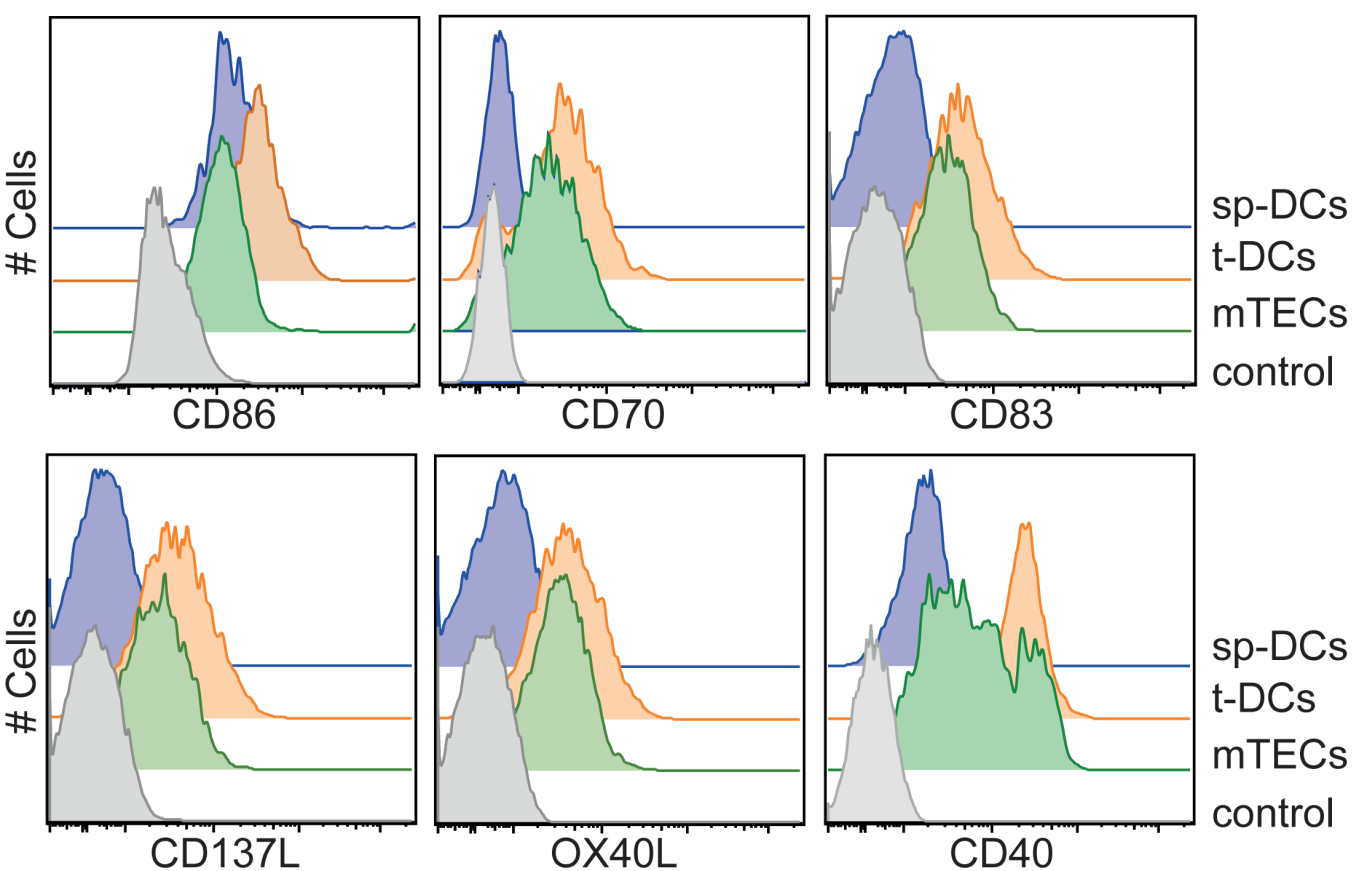
Expression of costimulatory molecules by thymic APCs APCs were enriched from thymi and spleens of BALB/c mice by enzymatic digestion and gradient centrifugation. Expression of CD86, CD70, CD83, CD137L, OX40L and CD40 was analyzed on gated CD45^−^EpCAM^+^Ly51^−^ mTECs, CD45^+^CD11c^hi^ Lin^−^ t-DCs and CD11c^hi^ Lin^−^ sp-DCs (Lin defined as CD90, CD49b, F4/80 and CD19) by flow cytometry. Gated CD4SP-Foxp3^−^ thymocytes were taken as control. Representative histograms from one out of four (CD86 and CD70), five (CD40) or three (CD83, CD137L and OX40L) independent experiments are depicted.

**Figure 4:**
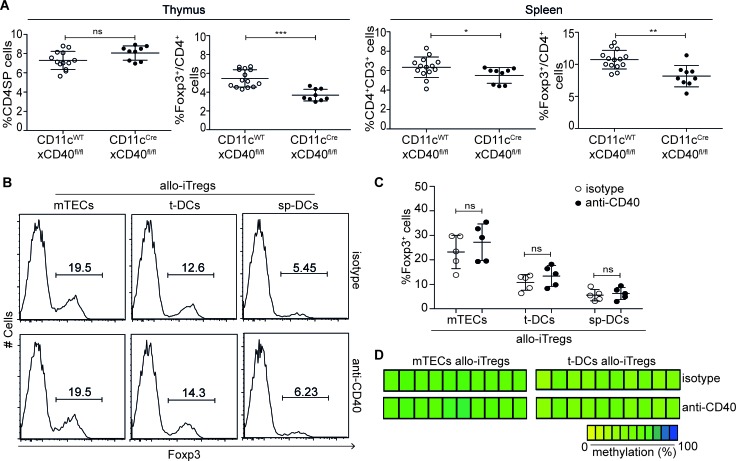
CD40-CD40L signaling is not critically required for generation of stable allo-iTregs **A.** Thymocytes and splenocytes from CD11c^Cre^xCD40^fl/fl^ (DC-specific CD40 knockout, filled circles) and CD11c^WT^xCD40^fl/fl^ mice (CD40 competent control, open circles) were analyzed by flow cytometry. Graphs show frequency of CD4SP thymocytes, Foxp3^+^ cells among CD4SP thymocytes, CD4^+^CD3^+^ splenocytes and Foxp3^+^ Tregs cells among CD4^+^CD3^+^ splenocytes. Data are summarized from two independent experiments (mean ± SD) and tested for significance using Mann-Whitney test; *p < 0.05; **p < 0.01; ***p < 0.001; ns, not significant. (B-D) Indicated APCs (BALB/c) were cultured with CD4SP-Foxp3^RFP−^ thymocytes (C57BL/6) in presence of IL-2 and either anti-CD40 or isotype control antibodies for six days. **B.** Expression of Foxp3 in cultured CD4^+^CD90^+^ thymocytes was analyzed by flow cytometry. Numbers indicate frequency of Foxp3^+^ cells. Representative data from one out of five independent experiments are depicted. **C.** Graph shows frequency of Foxp3^+^ allo-iTregs from cultures with indicated APCs in presence of anti-CD40 (filled circles) or isotype control antibodies (open circles). Data are summarized from five independent experiments (mean ± SD) and tested for significance using Mann-Whitney test; ns, not significant. **D.** At day 6, Foxp3^RFP+^ allo-iTregs were sorted from indicated cultures, and genomic DNA isolated from these cells was analyzed for the methylation status of TSDR. Representative data from one out of three independent experiments are depicted. Each bar represents one CpG motif. The degree of methylation at each CpG motif is represented according to the color code.

To identify molecular factors within thymic APCs that could contribute to the engraving of specific epigenetic signatures in developing Tregs, we first focused on the differentially expressed costimulatory molecules. Flow cytometric analysis of *ex vivo* isolated APCs revealed a substantial expression of CD86 on all APCs, with slightly higher levels found on t-DCs (Figure 3). Interestingly, CD70, CD83, CD137L (*Tnfsf9*) and OX40L (*Tnfsf4*) were expressed at higher levels on thymic APCs when compared to sp-DCs, with again t-DCs presenting highest levels. Similarly, high CD40 expression was observed for t-DC and a subset of mTECs, while sp-DCs expressed only low levels of CD40. CD40-CD40L signaling had been reported previously to influence thymic Treg development [31], however the specific contribution of CD40 expression on DCs for the *in vivo* generation of thymic Tregs had not been addressed so far. Hence, we here generated DC-specific CD40 knockout mice by crossing conditional CD40 knockout mice (N.G. and E. L., unpublished) to CD11c^Cre^ mice. In these mice (CD11c^Cre^xCD40^fl/fl^), we observed a mild, but significantly reduced frequency of Foxp3^+^ Tregs both in thymus and spleen when compared to CD11c^WT^xCD40^fl/fl^ mice taken as controls (Figure 4A), proving that CD40 expression on DCs is critical for the *in vivo* generation and/or homeostasis of thymic Foxp3^+^ Tregs. To study the contribution of CD40-CD40L signaling to the efficient induction of allo-iTregs by thymic APCs, we next performed *in vitro* alloantigen-specific Treg induction cultures using mTECs, t-DCs and sp-DCs, and tested the impact of antibodies blocking CD40-CD40L signaling on the frequency of Foxp3^+^ allo-iTregs. For all conditions tested, no significant differences in the frequency of Foxp3^+^ allo-iTregs were observed upon addition of anti-CD40 as compared to isotype control antibodies (Figure 4B+4C). Importantly, interference with CD40-CD40L signaling also had no impact on the TSDR methylation status in Foxp3^+^ allo-iTregs sorted from the corresponding cultures (Figure 4D). Although these findings suggest that CD40-CD40L signaling is not critically required for the efficient induction of stable Foxp3^+^ allo-iTregs by thymic APCs, it cannot be ruled out that mTECs and particularly t-DCs expressing high levels of CD40 had been conditioned *in vivo* upon receipt of signals via CD40 after encounter with CD40L^+^ cells [39]. To study the importance of thymic APC licensing via CD40, we first temporally abrogated CD40-CD40L signaling *in vivo* by injecting blocking anti-CD40L into Foxp3^GFPCre^ROSA26^RFP^ fate-mapping mice [40] for a period of nine days. However, this treatment did not result in any increase in exFoxp3^+^ cells (Supplementary Figure S5A, S5B). Next, we performed *in vitro* alloantigen-specific Treg induction cultures using t-DCs from the aforementioned DC-specific CD40 knockout mice (CD11c^Cre^xCD40^fl/fl^) as these DCs are deprived of any *in vivo* conditioning via CD40. Interestingly, we could not observe any difference in the frequency of Foxp3^+^ allo-iTregs between cultures using CD40-deficient and -proficient t-DCs (Supplementary Figure S5C), and preliminary results indicate a comparable demethylation at TSDR in Foxp3^+^ allo-iTregs sorted from the corresponding cultures (data not shown). Together, these findings indicate that licensing of thymic APCs via CD40 is not essential for the acquisition of their preferential ability to efficiently induce and stabilize Foxp3 expression in developing thymic Tregs.

In a next step, we investigated the functional role of other costimulatory molecules that were differentially expressed on mTECs and t-DCs compared to sp-DCs by performing *in vitro* alloantigen-specific Treg induction cultures in presence of blocking antibodies. Neither addition of anti-CD70, anti-CD137L and anti-OX40L alone or combined blockade of various costimulatory molecules (CD40, CD70, CD137L and OX40L) resulted in a decreased frequency of Foxp3^+^ allo-iTregs when compared to control cultures (Supplementary Figure S6A). Furthermore, when alloantigen-specific Treg induction cultures were performed up with APCs isolated from either CD83-mutant or CD83-transgenic mice, no differences in the frequency of Foxp3^+^ allo-iTregs compared to cultures with APCs isolated from WT control mice were observed (Supplementary Figure S6B). Finally, we investigated the role of soluble factors secreted by thymic APCs. For this purpose, we generated conditioned media by culturing *ex vivo* isolated mTECs, t-DCs and sp-DCs together with allogeneic CD4SP Foxp3^−^ thymocytes for 48h. Addition of these conditioned media to ‘fresh’ alloantigen-specific Treg induction cultures did not significantly affect the frequency of Foxp3^+^ Tregs induced in any of the cultures (Supplementary Figure S6C). Together, these data ruled out any significant contribution of major costimulatory molecules expressed on thymic APCs and secretory factors released by them, in the generation of stable alloantigen-specific Foxp3^+^ Tregs.

### Phenotype and *in vitro* suppressive properties of thymic APC-induced allo-iTregs

**Figure 5:**
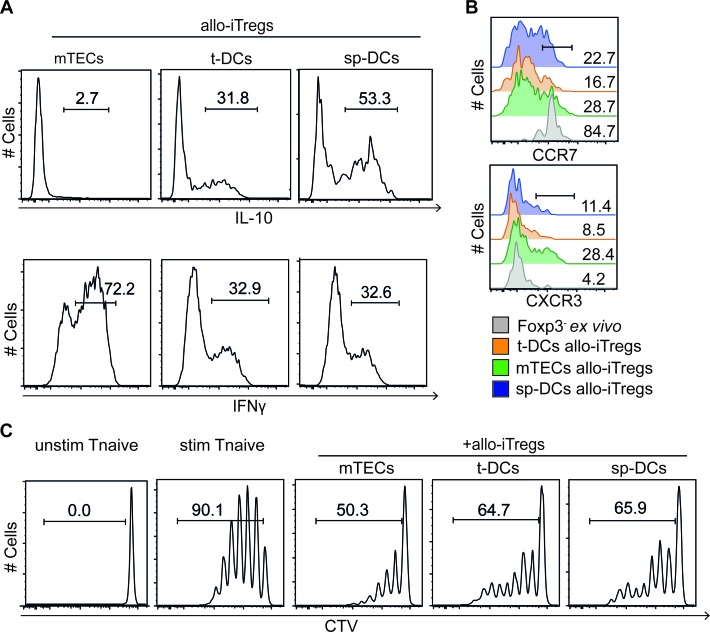
*In vitro* phenotypic and functional characterization of allo-iTregs generated by thymic APCs Indicated APCs (BALB/c) were cultured with CD4SP Foxp3^RFP−^ thymocytes (C57BL/6) in presence of IL-2 for six days. **A.** At day 6, cells were restimulated with phorbol 12-myristate 13-acetate and ionomycin for detection of IFN-γ, and expression of IL-10^GFP^ (top) and IFN-γ (bottom) was assessed by flow cytometry in gated Foxp3^RFP+^ allo-iTregs from indicated cultures. Numbers indicate frequency of IL-10^+^ and IFN-γ^+^ cells. Representative data from one out of four independent experiments are depicted. **B.** At day 6, expression of CCR7 (top) and CXCR3 (bottom) was directly assessed by flow cytometry on gated Foxp3^RFP+^ allo-iTregs from indicated cultures. *Ex vivo* isolated CD4SP Foxp3^−^ thymocytes served as controls. Numbers indicate frequency of CCR7^+^ and CXCR3^+^ cells. Data were taken from one out of two independent experiments. **C.** At day 6, Foxp3^RFP+^ allo-iTregs were sorted from indicated cultures by flow cytometry. Freshly isolated, CTV-labeled naïve CD4^+^ T cells were stimulated with anti-CD3/anti-CD28 beads in presence of indicated allo-iTregs at a ratio of 1:4 (Tregs to naïve T cells). Naïve T cells stimulated in absence of allo-iTregs (stim Tnaive) as well as naïve T cells receiving no stimulus (unstim Tnaive) served as controls. After four days, proliferation of naïve T cells was assessed by measuring CTV dilution in living naïve CD4^+^CD90.2^+^CD45.1^+^ T cells by flow cytometry. Numbers indicate frequency of cells in indicated gates. Representative data from one out of four independent experiments are depicted.

The interesting finding of thymic APCs being prime cellular players in initiating the demethylation at Treg-specific epigenetic signature genes in allo-iTregs, prompted us to phenotypically and functionally characterize these allo-iTregs. First, expression of IL-10 and IFN-γ, two cytokines known to play an important role for the suppressive activity of Foxp3^+^ Tregs [41], was analyzed. While mTEC-induced allo-iTregs completely lacked IL-10 expression, a significant fraction of t-DC-induced allo-iTregs expressed IL-10, comparable to sp-DC-induced allo-iTregs (Figure 5A and Supplementary Figure S7A). On the contrary, mTEC-induced allo-iTregs showed a significantly enhanced expression of IFN-γ when compared to both t-DC- and sp-DC- induced allo-iTregs. Although a similar cytokine expression profile was observed in corresponding Foxp3^−^CD4^+^ T cells from the same alloantigen-specific cocultures (Supplementary Figure S7B, S7C), we could not detect any difference in the expression of IL-10 and IFN-γ within *ex vivo* isolated Foxp3^+^ Tregs from thymus and spleen (Supplementary Figure S7D), suggesting that the abovementioned cytokine expression patterns were preferentially induced in the alloantigen-specific cultures. Next, expression of chemokine receptors CCR7 and CXCR3 was determined on the different allo-iTregs to assess their recirculating and inflammation-seeking capacity, respectively [42]. No differences in CCR7 expression were observed between mTEC-, t-DC- and sp-DC-induced allo-iTregs, while mTEC-induced allo-iTregs showed a higher frequency of CXCR3^+^ cells when compared to both DC-induced allo-iTregs (Figure 5B and Supplementary Figure S7E). Finally, we tested mTEC-, t-DC- and sp-DC-induced allo-iTregs in an *in vitro* suppression assay and observed a comparable suppressive capacity for all allo-iTregs (Figure 5C and Supplementary Figure S7F), suggesting that the differences in the methylation status at Treg-specific epigenetic signature genes do not affect *in vitro* suppressive function.

### Thymic APC-induced allo-iTregs delay skin graft rejection

**Figure 6:**
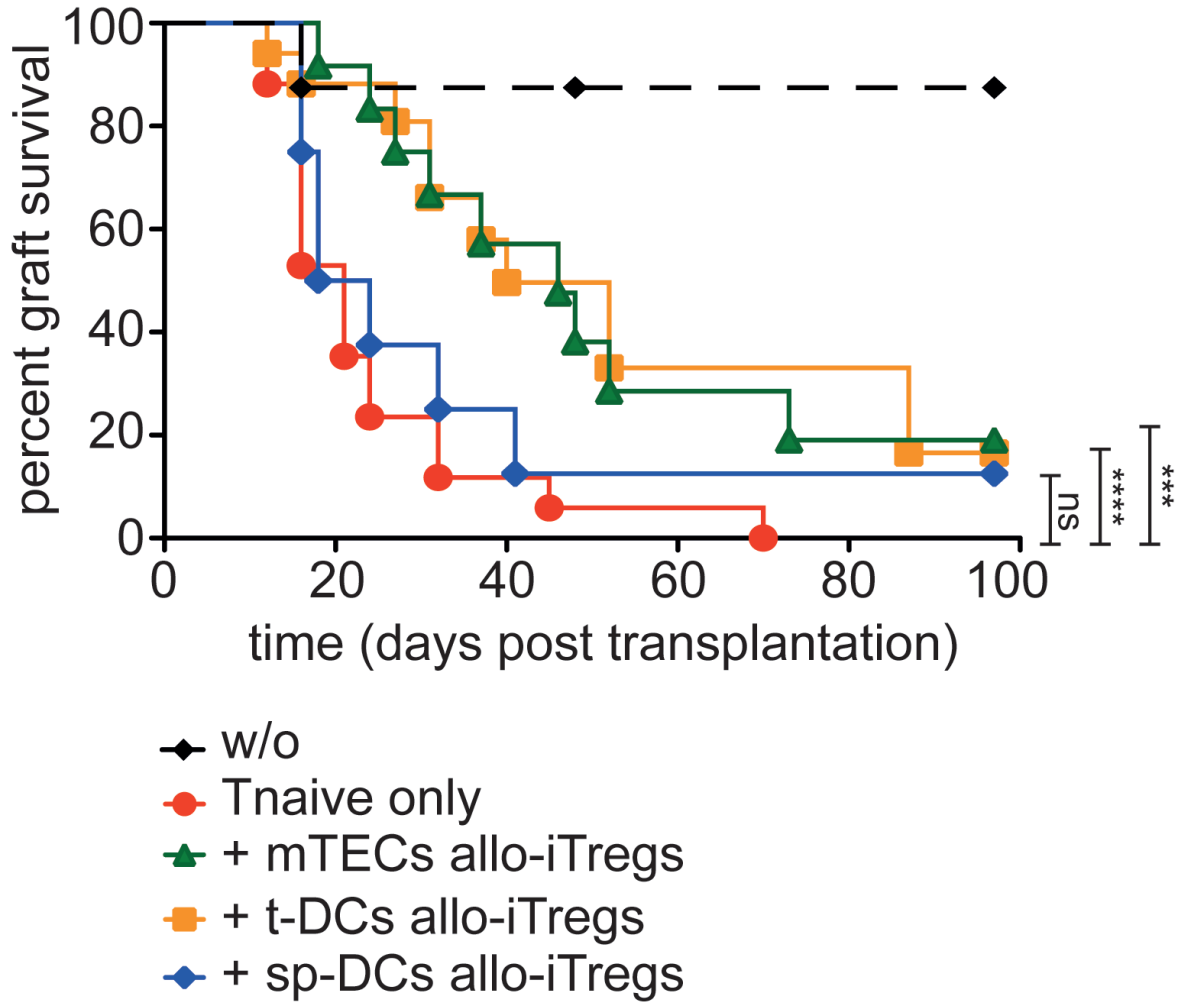
Allo-iTregs generated by thymic APCs very efficiently suppress skin allograft rejection Indicated APCs (BALB/c) were cultured with CD4SP Foxp3^RFP−^ thymocytes (C57BL/6) in presence of IL-2 for six days. One day prior to transplantation, allo-iTregs from indicated cultures were sorted by flow cytometry and injected into *Rag2^−/−^* mice. Mice were additionally injected with CD4^+^CD90.2^+^CD45.1^+^CD25^−^CD44^−^CD62L^hi^ naïve T cells. Mice receiving no cells (w/o) or naïve T cells only served as controls. One day later, allogeneic skin transplantation was performed. The following days animals were monitored for signs of rejection until day 100. Relative graft survival for each group is depicted and data are cumulative of three independent experiments; w/o (*n* = 6, black diamond), Tnaive only (*n* = 13, red circle), + mTEC allo-iTregs (*n* = 11, green triangle), + t-DC allo-iTregs (*n* = 12, orange square) and + sp-DC allo-iTregs (*n* = 8, blue diamond). Significance of survival graph was calculated between mice receiving Tnaive only versus mice co-transferred with different APC-induced allo-iTregs using log-rank (mantel cox) test. ***p < 0.001; ****p < 0.0001; ns, not significant.

Previous studies have demonstrated that iTregs although being functional *in vitro* lack suppressive potential when tested *in vivo*, a finding that was explained by the fully methylated Treg-specific epigenetic signature genes and their instable phenotype [11, 13]. Thus, we here tested the *in vivo* suppressive capacity of thymic APC-induced allo-iTregs, which show a pronounced demethylation at the TSDR and other Treg-specific epigenetic signature genes, in a highly immunogenic, allogeneic skin transplantation model. Thereto, allo-iTregs together with congenically marked (CD45.1) CD4^+^ naïve T cells were adoptively transferred into Rag2^−/−^ (C57BL/6) mice, one day prior to the skin transplant from BALB/c mice (Supplementary Figure S8A). Mice receiving CD4^+^ naïve T cells only or receiving no cells served as graft rejection or graft survival controls, respectively. Rapamycin was given to all groups on day -1, day 0 and day 2 in order to moderately suppress immediate triggering of the alloresponse. Graft survival was tracked until day 100 and the time point of graft rejection or day 100 in case of graft survival defined the end point. As expected, the group receiving only CD4^+^ naïve T cells rejected the skin graft and mice receiving no cells accepted the graft (Figure 6). Interestingly, allo-iTregs induced by both mTECs and t-DCs significantly improved graft survival in comparison to the group receiving CD4^+^ naïve T cells only. In contrast, sp-DC-induced allo-iTregs were incapable of significantly prolonging allograft survival (Figure 6), showing that only thymic APC-generated allo-iTregs display suppressive potential *in vivo*.

When we analyzed frequency and total cell number of adoptively transferred CD4^+^ naïve T cells and allo-iTregs at the individual end points within lymph nodes draining the skin transplantation site, we did not observe overt differences between the different groups (Supplementary Figure S8B-D). Furthermore, no differences could be detected regarding the frequency of *de novo* induced Foxp3^+^ Tregs among adoptively transferred CD4^+^ naïve T cells (Supplementary Figure S8E). However, we observed mild differences in the stability of Foxp3 expression among adoptively transferred allo-iTregs as both mTEC- and t-DC-induced allo-iTregs showed a slightly increased stability when compared to sp-DC-induced allo-iTregs (Supplementary Figure S8F). Taken together, these observations indicate that alloreactive Tregs induced by thymic APCs show a trend of higher maintenance of Foxp3 expression *in vivo* and can significantly prolong skin graft survival.

## DISCUSSION

Solid organ transplantation remains the only treatment option for several end-stage organ failures of heart, lung, kidney and liver; it yet comes with a hitch, graft rejection and side effects of unspecific immunosuppression. Immune cells of the organ recipient recognize and attack the graft as foreign, due to donor-recipient mismatch of major and minor histocompatibility complex antigens. Lately, use of immunosuppressive Tregs as cellular therapy has gained increasing interest to promote long-term graft survival by induction of immune tolerance [14, 43, 44]. However, the proposed clinical trials using polyclonal Tregs require cells in excess to efficiently suppress graft rejection [45], this can in turn result in increased immunosuppression and thereby increases the risk of infections. Thus, current concepts favor the use of alloantigen-specific Foxp3^+^ Tregs since the higher specificity of such allo-Tregs will reduce the number of Tregs required for injection into patients to promote graft survival. Hence, an accumulating body of knowledge about the benefits of using allo-Tregs to attain transplantation tolerance makes them promising candidates for therapeutic use [2, 46–48]. On the obverse side of the coin, there are still limitations in identifying stable allo-Tregs *ex vivo*. In addition, lack of standard protocols for *in vitro* generation of stable Tregs with alloantigen-specificity is a further major drawback. This is due to transient upregulation of Foxp3 expression by conventional T cells upon their activation or due to downregulation of Foxp3 expression in Tregs under certain inflammatory conditions [40, 49]. Therefore, the present study aimed at identifying cellular players and molecular mediators involved in Treg cell fate determination and maintenance, which could enable efficient *in vitro* generation of allo-Tregs with a stable phenotype.

We could demonstrate that allo-iTregs induced by thymic APCs show an increased efficacy in delaying skin graft rejection, whereas allo-iTregs induced by sp-DCs failed to prolong graft acceptance. Keeping feasible numbers in mind, low amounts of allo-iTregs were used in this preclinical model. Though the graft could not be fully rescued due to the high immunogenicity of the skin transplant, these numbers were enough to prolong graft acceptance by thymic APC-induced allo-iTregs. Two recent reports are supporting our finding, where one showed that Nrp1^+^ Tregs generated in the thymus could suppress skin graft rejection and another stated human thymus to be an important source of stable and suppressive Foxp3^+^ Tregs for clinical therapeutics [50, 51].

Although thymic APC-induced allo-iTregs showed the highest maintenance of Foxp3 expression upon *in vivo* transfer, a substantial fraction of allo-iTregs induced by sp-DCs could also retain Foxp3 expression. In accordance with our recent findings [13, 18, 19], these data suggest that mere Foxp3 expression is not sufficient to confer full functional competence to Tregs. Epigenetic remodeling of not only the *Foxp3* locus, but also of other Treg-specific epigenetic signature genes like *Eos*, *Ctla4* and *Gitr* is of utmost importance for the acquisition of their full suppressive capacity. Eos, CTLA-4 and GITR have been reported to be associated with stability, functional capacity and thymic differentiation of Tregs [52–55], and demethylation of these Treg-specific genes supports maintenance of their expression, being critical to establish stable Treg functional properties. Thus, the higher efficiency of mTECs and t-DCs as compared to sp-DCs to induce an epigenetic remodeling at *Eos*, *Ctla4* and *Gitr* might be causative for the superior *in vivo* suppressive capacity of thymic APC-induced allo-iTregs. It is worth mentioning that sp-DC-induced allo-iTregs also displayed a substantial demethylation at Treg-specific epigenetic signature genes, albeit at lower levels when compared to thymic APC-induced allo-iTregs, suggesting that also sp-DCs have the ability to induce epigenetic remodeling. The induction of Foxp3 expression and the formation of a Treg-specific epigenome are two independent events [21], which depend on the strength of TCR signaling and on the duration of TCR engagement [13]. Thus, it can be hypothesized that the lower demethylation at Treg-specific genes in sp-DC-induced Tregs could be an outcome of the reduced duration of TCR engagement by sp-DCs. We also observed a higher potential of thymic APCs to induce a high frequency of allo-iTregs, further supporting the essential role of thymic APCs for the generation of organ-specific Tregs within the thymus [56] and suggesting that also differences in the TCR repertoires of allo-iTregs induced by mTECs, t-DCs and sp-DCs might contribute to their *in vivo* functional properties [57]. Together, our findings indicate that thymic APCs express unique characteristics supporting both the induction and fate determination of Foxp3^+^ Tregs.

Additional to initiating the described epigenetic remodeling in allo-iTregs by thymic APCs, expression of both IL-10 and IFN-γ by t-DC-induced allo-iTregs and primarily expression of IFN-γ by mTEC-induced allo-iTregs was also observed. Both cytokines are associated with preventing allogeneic skin graft rejection and inhibiting GvHD progression [58–60]. Increased CXCR3 expression mainly by mTEC-induced allo-iTregs could be a result of high IFN-γ expression by these cells, which can induce CXCR3 expression in Tregs, and can effectively suppress allogeneic graft rejection [61, 62]. Since post-positive selection thymocytes require CCR7 for cortico-medullary migration, Foxp3^−^ thymocytes used for allo-iTreg generation *in vitro* were largely CCR7^+^. The comparable expression of CCR7 by the different allo-iTregs is hence likely to reflect their shared developmental stage, rather than shared functional characteristics. Although we cannot exclude that IL-10, IFN-γ, CCR7 and CXCR3 play a critical role for the suppression of skin graft rejection by allo-iTregs, they cannot account for the different *in vivo* suppressive properties observed for t-DC- and sp-DC-induced allo-iTregs as both allo-iTregs showed largely comparable cytokine and chemokine receptor expression patterns.

Gene expression profiling of APCs revealed highest differential gene expression by mTECs in comparison to DCs, possibly due to promiscuous tissue-specific gene expression by mTECs [63]. In addition, their shared developmental origin and migration of peripheral DCs into the thymus may account for the relative similarity of t-DCs and sp-DCs transcriptional profiles [64]. Many of the DEGs could be involved in the higher potency of thymic APCs in driving differentiation of a stable Treg lineage. The *in vivo* analysis of DC-specific CD40 knockout mice clearly indicated that CD40 expression on DCs is critical for the *in vivo* generation and/or homeostasis of thymic Foxp3^+^ Tregs, expanding our previous knowledge on the impact of CD40-CD40L signaling for the generation of tTregs [31, 65]. However, we could provide experimental evidence that licensing of thymic APCs via CD40 is not essential for the acquisition of their preferential ability to efficiently induce and stabilize Foxp3 expression in developing thymic Tregs. Furthermore, neither CD40-CD40L signaling, signals delivered through other major costimulatory molecules on thymic APCs nor secretory factors released by them seemed to be essential for the generation of stable alloantigen-specific Foxp3^+^ Tregs. Thus, the molecular factors within thymic APCs that could contribute to the engraving of specific epigenetic signatures in developing Tregs remain to be identified. These findings will be instrumental in directing efficient use of *in vitro* generated stable Tregs in therapeutic restoration of tolerance in clinical transplantation and autoimmune diseases.

## MATERIALS AND METHODS

### Animals

BALB/c mice and *Rag2*^−/−^ mice were purchased from Janvier and Jackson, respectively. *Foxp3^RFP^ x IL-10^GFP^* double reporter mice (C57BL/6 background) [66, 67], kindly provided by Richard Flavell (Yale University School of Medicine, New Haven, USA), C57BL/6 x CD45.1 mice, Foxp3^hCD2^ mice (BALB/c background) and mice with a DC-specific deletion of CD40 were bred at the animal facility of the Helmholtz Centre for Infection Research (Braunschweig, Germany). The conditional CD40 knockout mice (N.G. and E.L., unpublished) contained loxP sites upstream of exon 2 and downstream of exon 3. Cre-mediated deletion of the two ‚floxed’ exons not only eliminates sequences encoding the first and second TNFR-type Cys-rich domains, but also induces a frameshift mutation, thereby creating a non-functional allele. To generate mice with a DC-specific CD40 deletion, *Cd40*^fl/fl^ mice were crossed with CD11c^Cre^ mice [68]. CD83 transgenic mice (CD83tg, C57BL/6 background), expressing the CD83 molecule under control of the MHCI promoter [69], and CD83 mutant mice (CD83^lcd4^, C57BL/6 background), harbouring a missense mutation in the last exon of CD83 [70], were both bred at the animal facility of the Bernhard-Nocht-Institute for Tropical Medicine (Hamburg, Germany). Foxp3^GFPCre^ROSA26^RFP^ fate-mapping mice [40] were bred at the animal facility of the RIKEN Center for Integrative Medical Sciences. *Rag2^−/−^* mice were used at the age of eight weeks at the time of experimental procedure. All other mice were used at the age of four to ten weeks. All mice were housed and handled under specific pathogen-free conditions under supervision of institutional animal welfare officers and in accordance with approved protocols from the Institutional Animal Care at RIKEN or good animal practice as defined by FELASA and the national animal welfare body GV-SOLAS. The animal protocol was approved by the Niedersächsisches Landesamt für Verbraucherschutz und Lebensmittelsicherheit: animal licensing committee permission no. 33.14-42502-04-10/0071. Animal experiments were performed in accordance with institutional, state, and federal guidelines. Animals were handled with appropriate care and welfare, and all efforts were made to minimize suffering.

### Antibodies and flow cytometry

Cell suspensions from lymphoid organs were stained with fluorochrome-conjugated anti-mouse CD3 (17A2), CD4 (RM4-5), CD8α (53-6.7), CD11c (N418), CD19 (6D5), CD25 (PC61.5), CD40 (1C10), CD44 (IM7), CD45 (30-F11), CD45.1 (A20), CD49b (DX5), CD62L (MEL-14), CD70 (FR70), CD83 (Michel-17), CD86 (GL1), CCR7 (4B12), CXCR3 (CXCR3-173), EpCAM (G8.8), F4/80 (BM8), Ly51 (6C3), Ly51 (FG35.4), CD90.1 (HIS51), CD90.2 (53-2.1), CD137L (TKS-1), CD252 (RM134L), TCRβ (H57-597) and anti-human CD2 (RPA-2.10) that were purchased from either BD Biosciences, Biolegend or eBioscience. Intracellular staining was performed with Foxp3 staining kit for Foxp3 (FJK-16s) and IFN-γ (XMG1.2) Abs according to manufacturer's instructions (eBioscience). For IFN-γ cytokine staining, cells were stimulated with phorbol 12-myristate 13-acetate (Sigma) and ionomycin (Sigma) for 4 h and with brefeldin A for the last 2 h. For dead cell exclusion LIVE/DEAD^®^ fixable Near-IR stain kit (Invitrogen) or Sytox Blue (Molecular Probes) was used. Flow cytometric analysis was performed on LSR II (BD Biosciences) and data were analyzed using FlowJo software (Tree Star).

### Purification of thymocytes and peripheral T cells

For purification of CD4SP thymocytes from male or female donor mice, total thymocytes were depleted of CD8^+^ cells using APC-conjugated anti-CD8^+^ and anti-APC microbeads (Miltenyi Biotec) followed by magnetic separation using the autoMACS separation system (Miltenyi Biotec). Peripheral CD4^+^ T cells were enriched from pooled spleen and lymph node cells using anti-CD4 microbeads followed by magnetic separation using the autoMACS separation system. CD4SP Foxp3^−^ thymocytes or CD4^+^Foxp3^−^ peripheral T cells were sorted as CD4^+^RFP^−^GFP^−^ cells from *Foxp3^RFP^ x IL-10^GFP^* reporter mice or stained with anti-human CD2 to isolate CD4SP Foxp3^hCD2−^ thymocytes from Foxp3^hCD2^ mice on FACS Aria II (BD Biosciences). For the *in vitro* and *in vivo* suppression assays, naïve CD4^+^ T cells were isolated from pooled spleen and lymph node cells from CD45.1 congenic C57BL/6 mice. Briefly, CD4^+^ T cells were enriched using anti-CD4 microbeads followed by magnetic separation using the autoMACS separation system. Subsequently, naïve CD4^+^ T cells (CD4^+^CD90.2^+^CD25^−^CD44^−^CD62L^hi^) were sorted on FACS Aria II.

### Purification of APCs

To isolate thymic APCs and sp-DCs, thymic lobes and spleens were separated from male or female mice, finely chopped into pieces and digested in complete RPMI 1640 medium (Life Technologies) containing 0.2 mg/ml collagenase/dispase (Roche), 0.25 mg/ml DNase I (Roche) and incubated at 37°C for 1 h. Liberated cells were filtered through 100 μM nylon mesh and subjected to percoll gradient using 1.115 g/ml high-density percoll and 1.06 g/ml of low-density percoll. The gradient was centrifuged at 1350 g for 30 min at 4°C. The low-density interface was collected for APCs. Cells were sorted as CD45^−^EpCAM^+^Ly51^−^ for mTECs, CD45^+^CD11c^hi^ Lin^−^ (Lin is defined as CD90, CD49b, F4/80 and CD19) for t-DCs and CD11c^hi^ Lin^−^ for sp-DCs on FACS Aria II.

### Cell culture

Cells were cultured in RPMI 1640 medium (Life Technologies) supplemented with penicillin (50 U/ml), streptomycin (50 U/ml), HEPES (25 mM), sodium pyruvate (1 mM), β-mercaptoethanol (50 μM) (all purchased from Biochrom) containing 10 % fetal calf serum (Sigma-Aldrich), at 37°C, 5 % CO_2_ in 96U bottom plates (Corning).

### *In vivo* blocking of CD40-CD40L signaling

Male or female Foxp3^GFPCre^ROSA26^RFP^ fate-mapping mice were repetitively injected *i.p*. with 0.3 mg anti-CD40L (MR1) in 300 μl PBS at days 0, 2, 4, 6 and 8. Control mice received PBS injections. At day 9, mice were sacrificed and the frequency of total Foxp3^GFP+^ as well as exFoxp3^+^ cells (GFP^−^RFP^+^) among CD4SP thymocytes and splenic CD4^+^ T cells was determined by flow cytometry.

### *In vitro* Treg differentiation assay

For the alloantigen-specific system, 10×10^4^ CD4^+^Foxp3^−^ cells derived from *Foxp3^RFP^ x IL-10^GFP^* double reporter mice (C57BL/6 background) or Foxp3^hCD2^ (BALB/c background) were plated with either mTECs (APC to T cell ratio 1:50 if not indicated otherwise) or DCs (APC to T cell ratio 1:10 if not indicated otherwise) in presence of 100 ng/ml recombinant mouse IL-2 (R&D systems) for six days. For *in vitro* blocking experiments, titrated amounts of anti-CD40 (HM40-3, Biolegend), anti-CD70 (FR70, Biolegend), anti-CD137 (TKS-1, Biolegend) and anti-OX40L (RM134L, Biolegend) was used. At the end of the cultures, allo-iTregs were either directly phenotypically analyzed or sorted as CD4^+^CD90^+^Foxp3^+^ cells for subsequent analyses on FACS Aria II using the Foxp3 reporter molecules RFP or hCD2.

### *In vitro* Treg suppression assay

Sorted allo-iTregs were cultured with freshly isolated, Cell Trace Violet (CTV)^TM^ (Invitrogen)-labeled naïve CD4^+^ T cells at ratios from 1:4 to 1:32 (Tregs to naïve T cells). Anti-CD3/anti-CD28 beads (Invitrogen) were used for polyclonal stimulation. After four days, proliferation of naïve T cells was assessed by measuring CTV dilution in living naïve CD4^+^CD90.2^+^CD45.1^+^ T cells by flow cytometry.

### Allogeneic skin transplantation and adoptive transfer of cells

To induce or prevent an immune reaction against the skin graft 2.5×10^5^ FACS-sorted peripheral naïve T cells (CD4^+^CD90.2^+^CD45.1^+^CD25^−^CD44^−^CD62L^hi^) isolated from female C57BL/6 mice were injected intravenously (*i.v*.) into female *Rag2-/−* mice (C57BL/6) with or without 5×10^5^ allo-iTregs (CD4^+^CD90.2^+^Foxp3^RFP+^). As control, PBS was *i.v* injected into *Rag2^−/−^* mice. The following day mice were anesthetized with an equal mixture of 2 % Rompun (Bayer) and 100 mg/ml Ketanest (Eurovet Animal Health B.V., 500 μl each) with 4.5 ml NaCl. 10 μl per g bodyweight were administered intraperitoneally (*i.p*.) into the mouse. Full thickness tail skin (about 1 cm^2;^ BALB/c) was grafted onto the lateral flank of the recipient *Rag2^−/−^* mouse. The wound was applied with an ointment and was protected firstly with an elastic bandage (Rancolast^®^, Lohmann&Rauscher). Then a soft tape (Leukopor^®^, BSN) was applied and finally the dressing was done using the surgical tape (Blenderm™, 3M). The whole bandage was carefully removed after 7-10 days. The graft was monitored every two days for rejection characteristics. Rapamycin (LC Laboratories) was injected *i.p*. three times at day -1, day 0 and day 2 at 90 μg/mouse. Mice were monitored for graft survival until day 100.

### RNA-Seq

Total RNA was isolated from purified APCs with RNAeasy Plus mini kit (Qiagen). Quality and integrity of total RNA was controlled on Agilent Technologies 2100 Bioanalyzer (Agilent Technologies). Purification of poly*-*A containing mRNA was done using poly*-*T oligo attached magnetic beads (Illumina). Following the purification, the mRNA was used for library preparation using Script Seq v2 Library preparation kit (Illumina). The sequencing was carried out on Illumina HiSeq2500 using 50 bp single read. The sequenced libraries were assessed for read quality with *FastQC* (http://www.bioinformatics.babraham.ac.uk/projects/fastqc). Quality assessment showed neither insufficient read quality, nor nucleotide frequency biases introduced by primer contamination. Therefore, libraries were directly aligned versus mouse reference genome (assembly: GRCm38) using splice junction mapper *Tophat2* v1.2.0 [71] with default parameterization. Supplementary Table S4 shows an overview of resulting mapping statistics.

Reads aligned to annotated genes were quantified with *htseq-count* (http://www-huber.embl.de/users/anders/HTSeq) program, and determined read counts served as input to *DESeq2* [72] for pairwise detection and quantification of differential gene expression between the three different conditions. In addition, RPKM (reads per kilobase max. transcript length per million mapped reads) values were computed for each library from raw gene counts and a PCA of the log_2_ transformed, scaled and mean centred RPKM values was performed using base functions *scale* and *prcomp* from the statistical data analysis framework *R*. Shown heatmaps were generated with R package *pheatmap*. The list of *DESeq2* determined differentially expressed genes (DEGs) was filtered with an absolute log_2_fold change (FC) cutoff of at least 1.5 and a *p*-value cutoff, corrected for multiple testing, of at most 0.05. This filtering results in the numbers of up-/down-regulated genes shown in Supplementary Table S1. RNA-Seq data can be accessed under GEO/SRA accession number GSE67834.

### Gene ontology and pathway enrichment analysis

The association of Gene Ontology (GO) terms and KEGG metabolic pathways to genes in the lists of DEGs (|log2FC|> = 1.5 and adjusted *p*-value< = 0.05) resulting from the pairwise comparisons mTECs vs. sp-DCs, mTECs vs. t-DCs and t-DCs vs. sp-DCs was assessed with functions from the R package GOstats [73]. For the applied conditional hypergeometric test for overrepresentation of GO terms in each of the three ontologies (molecular function, biological process and cellular component) and annotated KEGG pathways we used a *p*-value cutoff of 0.001. The used GO annotations were obtained from the Bioconductor *Mus musculus* annotation package, whereas KEGG pathway annotations were directly retrieved from KEGG using KEGG‘s REST API.

### Methylation analysis

For all methylation analyses, cells from male mice were used. Genomic DNA was isolated from purified cells with the NucleoSpin Tissue XS kit (Macherey-Nagel) following the manufacturer's recommendations. Purified DNA was quantified by measuring the absorption of light at 260 nm wavelength with a Nanodrop 1000 spectrophotometer (Peqlab). For analysis of TSDR, the TSDR was amplified by PCR containing 10 ng of bisulfite-converted genomic DNA, HotStar Taq PCR buffer (Qiagen), 1 U HotStar Taq DNA polymerase, 2.5 mM MgCl_2_ and 0.38 μM each of forward and reverse primers in a final volume of 50 μl (Cycle: 95°C for 15 min; 50x 95°C for 30 sec, 57°C for 1 min, 72°C for 1 min; 72°C for 7 min). The PCR product was analyzed by gel electrophoresis. 20-40 μl of the PCR product, Pyromark Gold Q96 reagents (Qiagen), Pyromark buffers (Qiagen), Streptavidin Sepharose (GE Healthcare) and sequencing primers (Supplementary Table S5) were used for pyrosequencing on a PSQ96MA (Qiagen) according to the manufacturer's protocol. For Treg-specific epigenetic signature genes, the analysis was carried out using bisulfite sequencing as described previously [13].

### Statistics

Calculations were performed using Graph Pad Prism v5.0 (Graph-Pad software). Most of the analyses were performed using nonparametric Mann-Whitney test (two-tailed, confidence intervals = 95 %) to compare groups and calculate *p*-values. Survival curves were calculated using Kaplan-Meier analysis and the *p*-values were analyzed with log-rank test (Mantel-Cox). A *p*-value of *p* < 0.05 was considered significant.

### Data and materials availability

The RNA-Seq data of the present study can be found via the following private access link:

http://www.ncbi.nlm.nih.gov/geo/query/acc.cgi?token=ozshogkqxrklfkz&acc=GSE67834

## SUPPLEMENTARY MATERIALS FIGURES AND TABLES












