# Supplementary material for: Unique properties of thymic antigen-presenting cells promote epigenetic imprinting of alloantigen-specific regulatory T cells
Source: Oncotarget. 2017 Mar 15;8(22):35542–57. doi: 10.18632/oncotarget.16221 (PMC5482597; doi:10.18632/oncotarget.16221)
Supplement: Supplementary file 1 [file oncotarget-08-35542-s001.pdf]

Unique properties of thymic antigen-presenting cells promote epigenetic imprinting of alloantigen-specific regulatory T cells

Supplementary Material

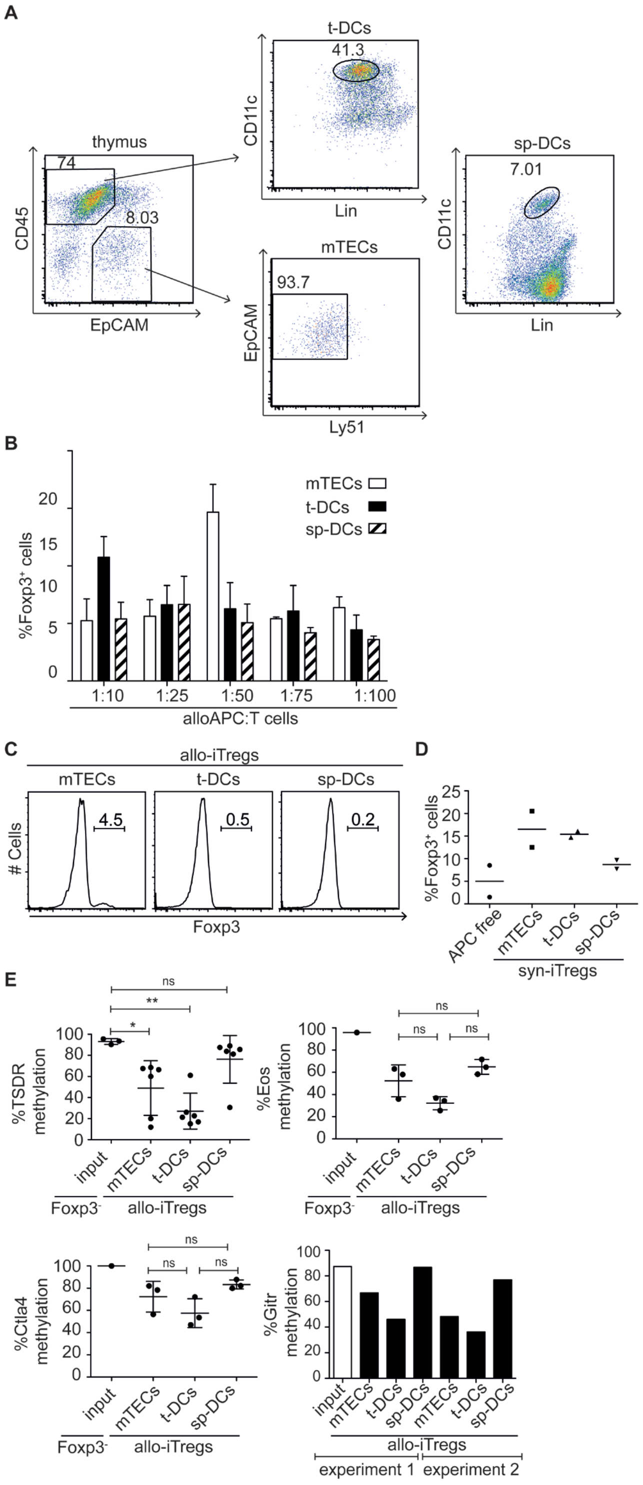

Supplementary Figure S1: Allo-iTreg generation and determination of the methylation status of TSDR and other Treg-specific epigenetic signature genes. (A) Flow cytometry sorting strategy for thymic APCs (mTECs and t-DCs; left/middle plots) and splenic DCs (sp-DCs; right plot). APCs, enriched from thymi and spleens of BALB/c mice by enzymatic digestion and gradient centrifugation, were sorted as $CD45^{-}EpCAM^{+}Ly51^{-}$ mTECs, $CD45^{+}CD11c^{hi}Lin^{-}$ t-DCs and $CD11c^{hi}$ Lin^{-} sp-DCs by flow cytometry (Lin defined as CD90, CD49b, F4/80 and CD19). Numbers indicate frequency of cells in corresponding gates. (B) mTECs (open bars), t-DCs (filled bars) and sp-DCs (hatched bars) all sorted from BALB/c mice were cultured with CD4SP $Foxp3^{RFP-}$ thymocytes (C57BL/6) at indicated ratios (ratio of APC to T cell) in presence of IL-2 for six days. Expression of Foxp3 was assessed in cultured $CD4^{+}CD90^{+}$ thymocytes by flow cytometry. Data are summarized from three independent experiments (mean ± SD). (C) Indicated APCs (BALB/c) were cultured with $CD4^{+}Foxp3^{RFP-}$ peripheral T cells isolated from spleen and lymph nodes (C57BL/6) in presence of IL-2 for six days (APC to T cell ratio 1:50 and 1:10 for mTECs and DCs, respectively). Expression of Foxp3 was assessed in cultured $CD4^{+}CD90^{+}$ T cells by flow cytometry. Numbers indicate frequency of $Foxp3^{+}$ cells. Data were taken from one out of two independent experiments. (D) CD4SP $Foxp3^{RFP-}$ thymocytes (C57BL/6) were either cultured alone or together with indicated syngeneic APCs (C57BL/6) in presence of IL-2 for six days. At day 6, expression of Foxp3 in cultured thymocytes was analyzed by flow cytometry. Graph shows frequency of $Foxp3^{+}$ syn-iTregs in gated $CD4^{+}CD90^{+}$ cells from indicated cultures. Data are summarized from two independent experiments. (E) At day 6, $Foxp3^{RFP+}$ allo-iTregs were sorted from indicated cultures, and genomic DNA isolated from these cells was analyzed for the methylation status of TSDR, *Eos*, *Ctla4* and *Gitr*. The average methylation status of all CpG motifs analyzed within respective epigenetic signature genes is depicted. Data are summarized from six (TSDR) or three (*Eos*, *Ctla4*) independent experiments (mean ± SD). For *Gitr,* results from two independent experiments are depicted. Genomic DNA from CD4SP $Foxp3^{-}$ thymocytes was analyzed as input control for the methylation status of the TSDR (three independent samples) and *Eos*, *Ctla4* and *Gitr* (unicate). For the methylation status of the TSDR, *Eos* and *Ctla4* significance was tested using Mann-Whitney test; * $p < 0.05$; ** $p < 0.01$; ns, not significant.

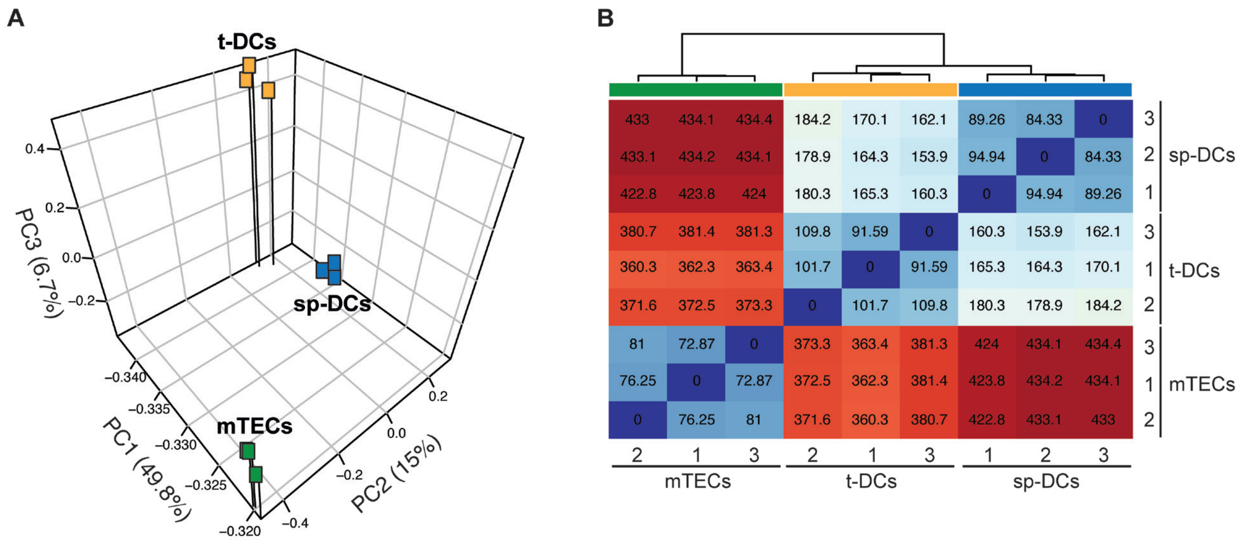

Supplementary Figure S2: Global analysis of RNA-Seq data from mTECs, t-DCs and sp-DCs. *Ex vivo* isolated mTECs, t-DCs and sp-DCs (all from BALB/c mice) were transcriptionally profiled by RNA-Seq in triplicates. (A) PCA of mean centered and scaled gene expression values (RPKM) of mTECs, t-DCs and sp-DCs. (B) Heatmap of Euclidian sample distances of *rlog*-transformed read counts for RNA-Seq data.

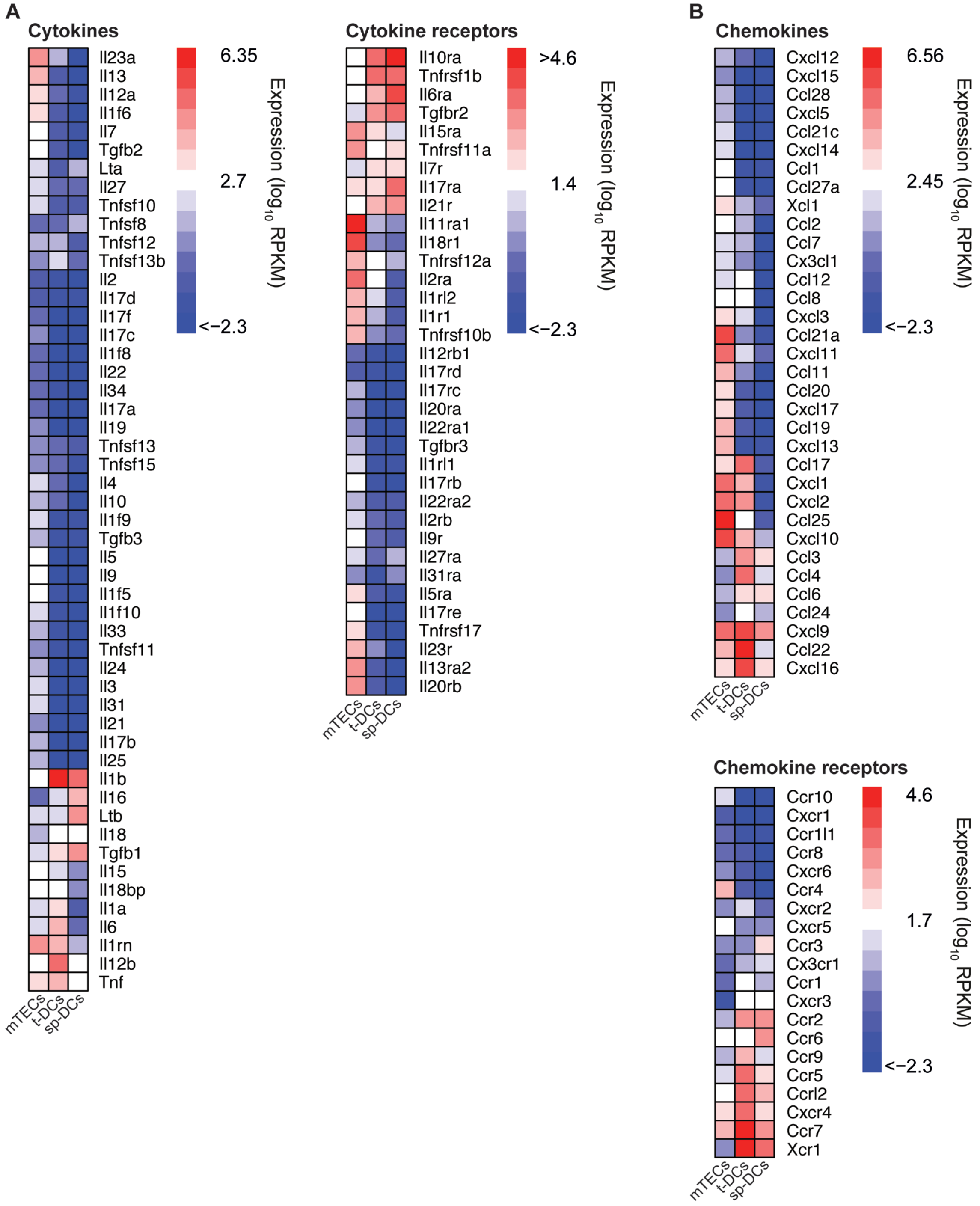

Supplementary Figure S3: Differential gene expression of cytokines/cytokine receptors and chemokines/chemokine receptors on APC subsets. *Ex vivo* isolated mTECs, t-DCs and sp-DCs were transcriptionally profiled by RNA-Seq. Heatmap of mean averaged over replicates RPKM normalized expression values of (A) cytokines/cytokine receptors and (B) chemokine/chemokine receptors on indicated APCs.

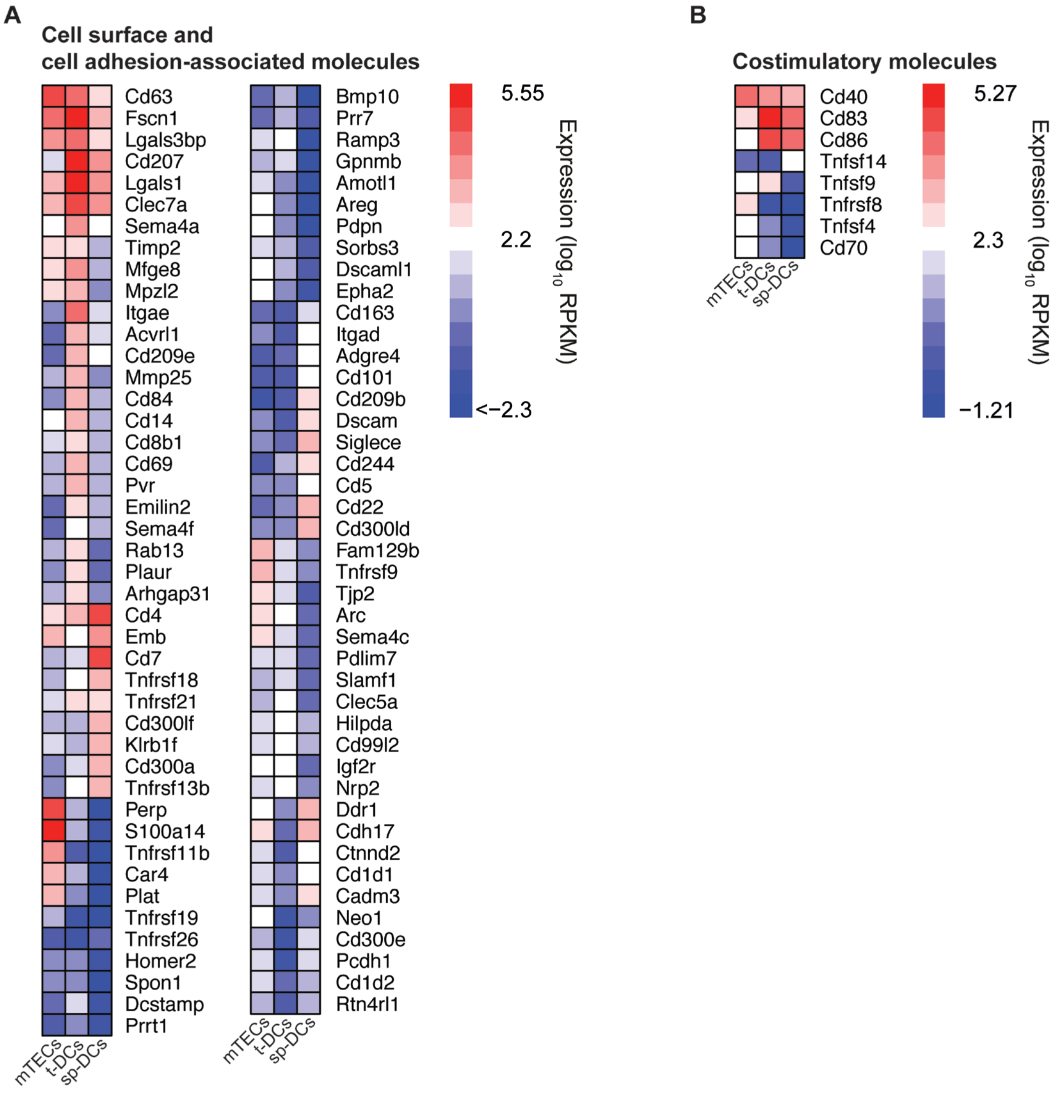

Supplementary Figure S4: Differential gene expression of cell surface/cell adhesion-associated molecules and costimulatory molecules on APC subsets. *Ex vivo* isolated mTECs, t-DCs and sp-DCs were transcriptionally profiled by RNA-Seq. Heatmap of mean averaged over replicates RPKM normalized expression values of (A) cell surface/cell adhesion-associated molecules and (B) costimulatory molecules on indicated APCs.

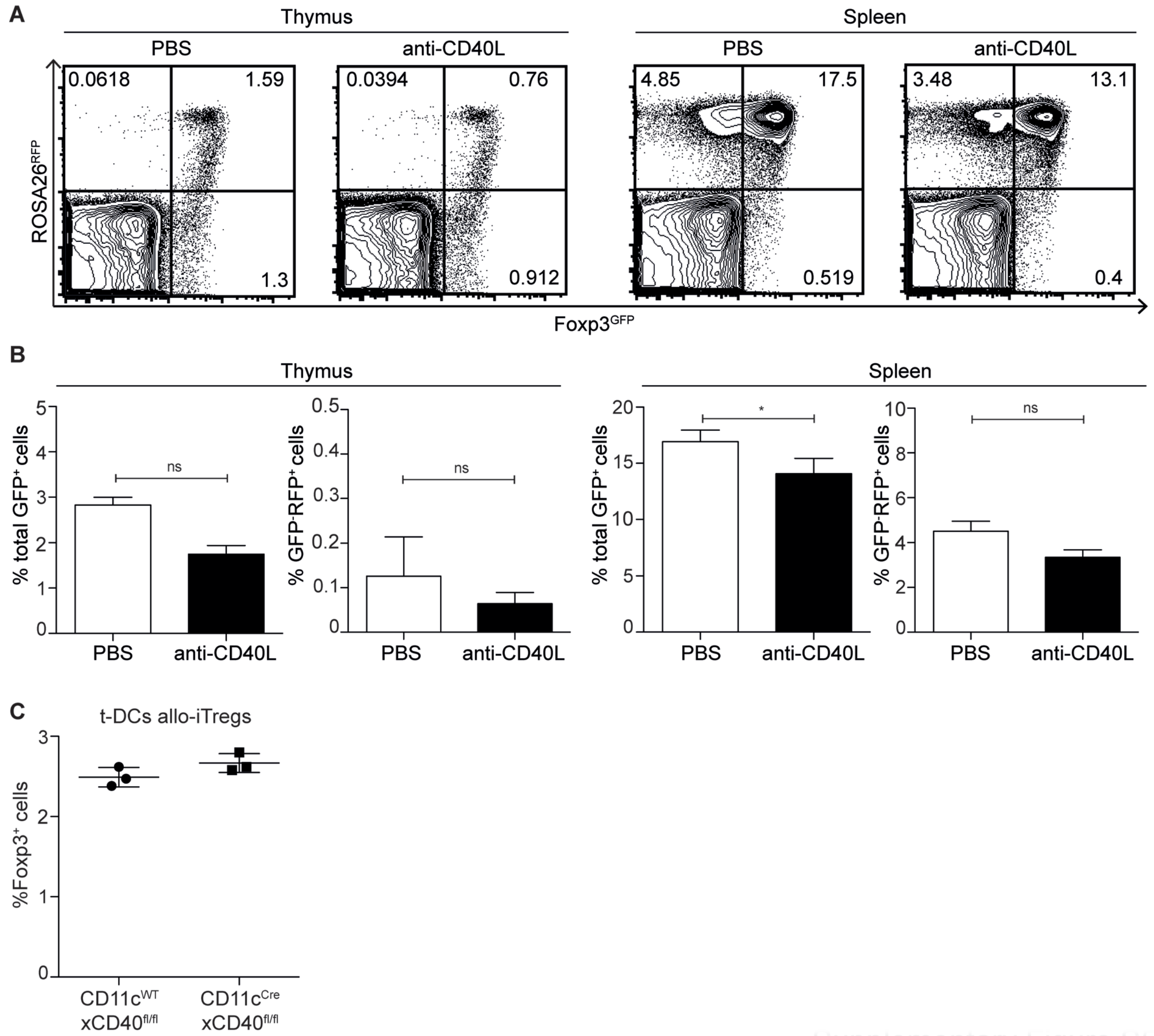

Supplementary Figure S5: CD40-CD40L signaling is not critically required for stabilization of Foxp3 expression *in vivo*. Foxp3^{GFPCre}ROSA26^{RFP} fate-mapping mice received repetitive *i.p.* injections (five times every other day) of anti-CD40L (MR1) or were treated with PBS as control. One day after the final antibody administration, frequency of total Foxp3^{GFP+} as well as exFoxp3^{+} cells (GFP^{-}RFP^{+}) among CD4SP thymocytes and splenic CD4^{+} T cells was determined by flow cytometry. (A) Representative dot plots from four anti-CD40L-treated or three PBS-treated mice are depicted. Cells were gated as CD4^{+}CD8^{-}TCRβ^{+}. Numbers indicate frequency of cells in quadrants. (B) Graphs show frequency of total Foxp3^{GFP+} as well as exFoxp3^{+} cells (GFP^{-}RFP^{+}) among CD4SP thymocytes (left) and splenic CD4^{+} T cells (right). Data are summarized from three to four individually treated mice (mean ± SD) and tested for significance using Mann-Whitney test; * $p < 0.05$; ns, not significant. (C) t-DCs, sorted from CD11c^{Cre}xCD40$^{fl/fl}$ (DC-specific CD40 knockout) or CD11c^{WT}xCD40$^{fl/fl}$ (CD40 competent control) mice (both C57BL/6 background), were cultured with CD4SP Foxp3^{hCD2-} thymocytes (BALB/c) in presence of IL-2 for six days. Expression of Foxp3 in cultured CD4^{+}CD90^{+} thymocytes was assessed by flow cytometry. Graph shows frequency of Foxp3^{+} allo-iTregs from indicated cultures. Technical triplicates (mean ± SD) from one out of two independent experiments are depicted.

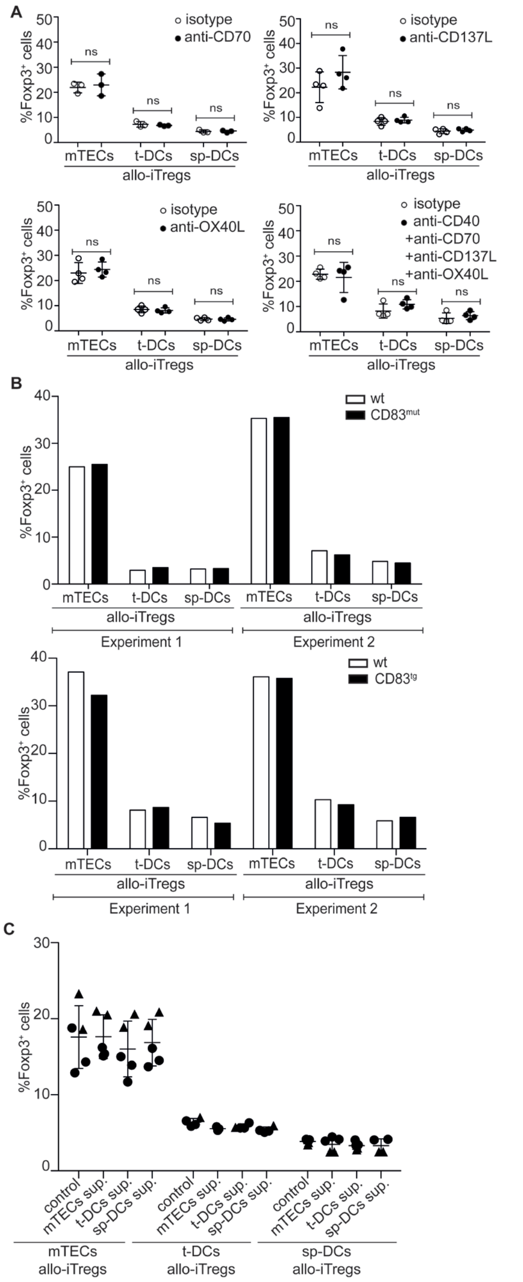

Supplementary Figure S6: Neither signaling via costimulatory pathways nor soluble factors derived from thymic APCs are critically required for induction of allo-iTregs. (A) Indicated APCs (BALB/c) were cultured with CD4SP Foxp3^{RFP-} thymocytes (C57BL/6) in presence of IL-2 for six days. Anti-CD70, anti-CD137L and anti-OX40L were added either separately or combined together with anti-CD40 from the beginning of the culture (filled circles). Isotype control antibodies were taken as controls (open circles). Expression of Foxp3 in cultured CD4$^+$CD90$^+$ thymocytes was analyzed by flow cytometry. Data are summarized from three (anti-CD70) and four (anti-CD137L, anti-OX40L, combined treatment) independent experiments (mean ± SD) and tested for significance using Mann-Whitney test; ns, not significant. (B) Indicated APCs isolated from CD83 mutant mice (top, filled bars) or CD83 transgenic mice (bottom, filled bars), both on C57BL/6 background, were cultured with CD4SP-Foxp3$^-$ thymocytes from Foxp3^{hCD2} (BALB/c background) in presence of IL-2 for six days. APCs isolated from C57BL/6 mice (open bars) were taken as controls (WT). Expression of Foxp3 in cultured CD4$^+$CD90$^+$ thymocytes was analyzed by flow cytometry. Graphs show frequency of Foxp3$^+$ allo-iTregs from two independent cultures. (C) Conditioned media from 48h-cultures of *ex vivo* isolated mTECs, t-DCs and sp-DCs (all BALB/c) with CD4SP-Foxp3^{RFP-} thymocytes (C57BL/6) was added to cocultures of indicated freshly isolated APCs (BALB/c) with CD4SP-Foxp3^{RFP-} thymocytes (C57BL/6) in presence of IL-2 for six days. Fresh medium was used as control. Expression of Foxp3 in cultured CD4$^+$CD90$^+$ thymocytes was analyzed by flow cytometry. Graph shows frequency of Foxp3$^+$ allo-iTregs from indicated cultures. Data are summarized (all technical replicates) from two independent experiments (mean ± SD).

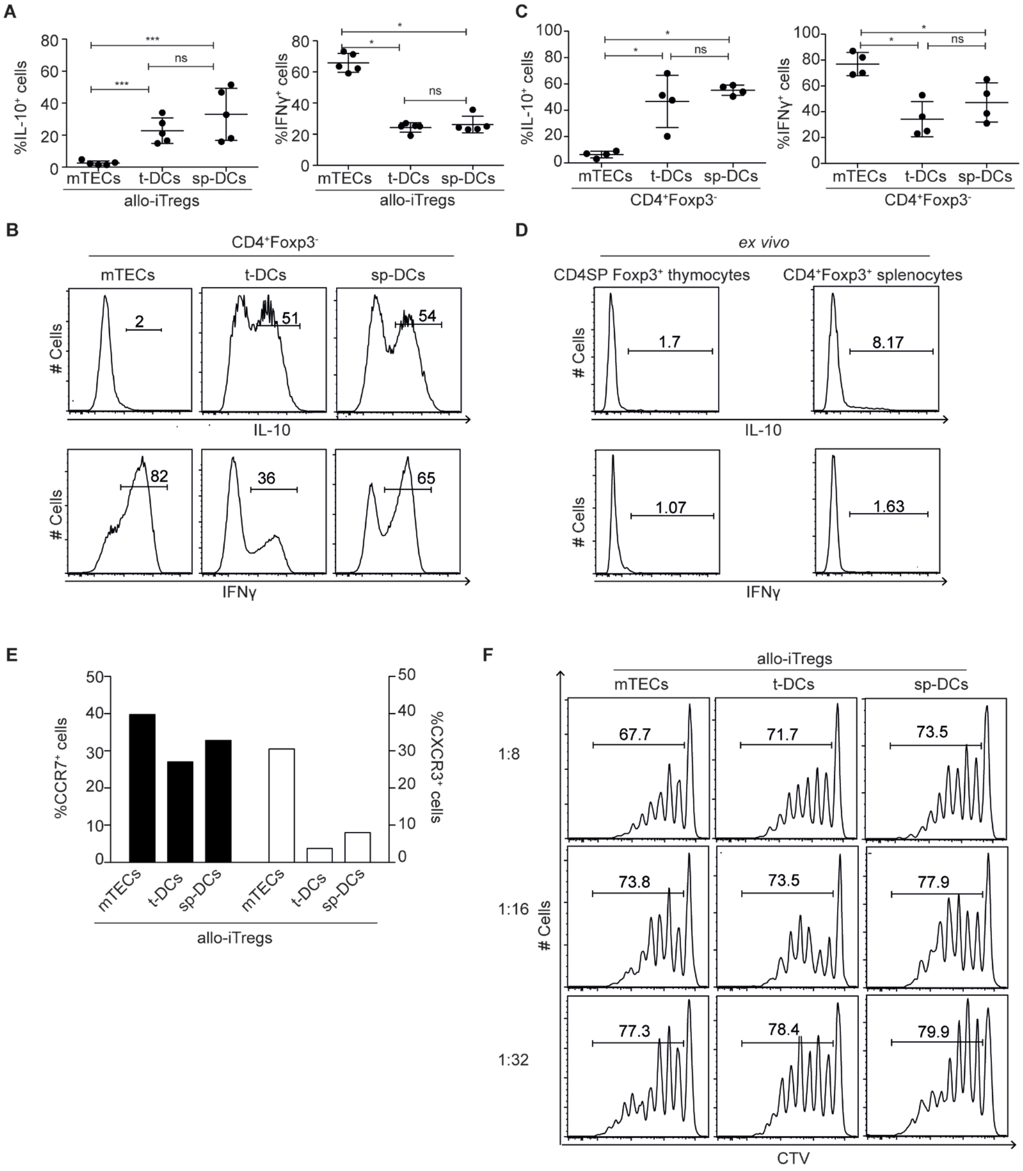

Supplementary Figure S7: *In vitro* phenotypic and functional characterization of Foxp3$^-$ and Foxp3$^+$ cells from allogeneic co-cultures. Indicated APCs (BALB/c) were cultured with CD4SP Foxp3^{RFP-} thymocytes (C57BL/6) in presence of IL-2 for six days. (A) At day 6, cells were restimulated with phorbol 12-myristate 13-acetate and ionomycin for detection of IFN-γ, and expression of IL-10^{GFP} (left) and IFN-γ (right) was assessed by flow cytometry in gated Foxp3^{RFP+} allo-iTregs from indicated cultures. Data are summarized from five independent experiments (mean ± SD) and tested for significance using Mann-Whitney test; * $p < 0.05$; *** $p < 0.001$; ns, not significant. (B-C) Cells were treated as described above, and expression of IL-10^{GFP} (upper row) and IFN-γ (lower row) was assessed by flow cytometry in gated Foxp3^{RFP-} T cells from indicated cultures. (B) Representative data from one out of four independent experiments are depicted. Numbers indicate frequency of IL-10$^+$ and IFN-γ$^+$ cells. (C) Graphs show frequency of IL-10$^+$ (top) and IFN-γ$^+$ cells (bottom) within gated Foxp3^{RFP-} cells from indicated cultures. Data are summarized from four independent experiments (mean ± SD) and tested for significance using Mann-Whitney test; * $p < 0.05$; ns, not significant. (D) *Ex vivo* isolated thymocytes and splenocytes from *Foxp3^{RFP} x IL-10^{GFP}* double reporter mice were tested for IL-10 expression by flow cytometry (upper row). For analysis of IFN-γ expression, *ex vivo* isolated thymocytes and splenocytes from BALB/c mice were stimulated with phorbol 12-myristate 13-acetate and ionomycin, followed by flow cytometry analysis (lower row). Histograms show frequency of cytokine$^+$ cells within gated CD4SP Foxp3$^+$ thymocytes and CD4$^+$Foxp3$^+$ splenocytes. Data from two independent experiments (IL-10) or two independently analyzed mice (IFN-γ) are depicted. (E) At day 6, expression of CCR7 (left, filled bars) and CXCR3 (right, open bars) was directly assessed by flow cytometry on gated Foxp3^{RFP+} allo-iTregs from indicated cultures. Data were taken from one out of two independent experiments. (F) At day 6, Foxp3^{RFP+} allo-iTregs were sorted from indicated cultures by flow cytometry. Freshly isolated, CTV-labeled naïve CD4$^+$ T cells were stimulated with anti-CD3/anti-CD28 beads in presence of indicated allo-iTregs at ratios of 1:8, 1:16 and 1:32 (Tregs to naïve T cells). After four days, proliferation of naïve T cells was assessed by measuring CTV dilution in living naïve CD4$^+$CD90.2$^+$CD45.1$^+$ T cells by flow cytometry. Numbers indicate frequency of cells in indicated gates. Data are representative of one out of three (1:16) or four (1:8 and 1:32) independent experiments.

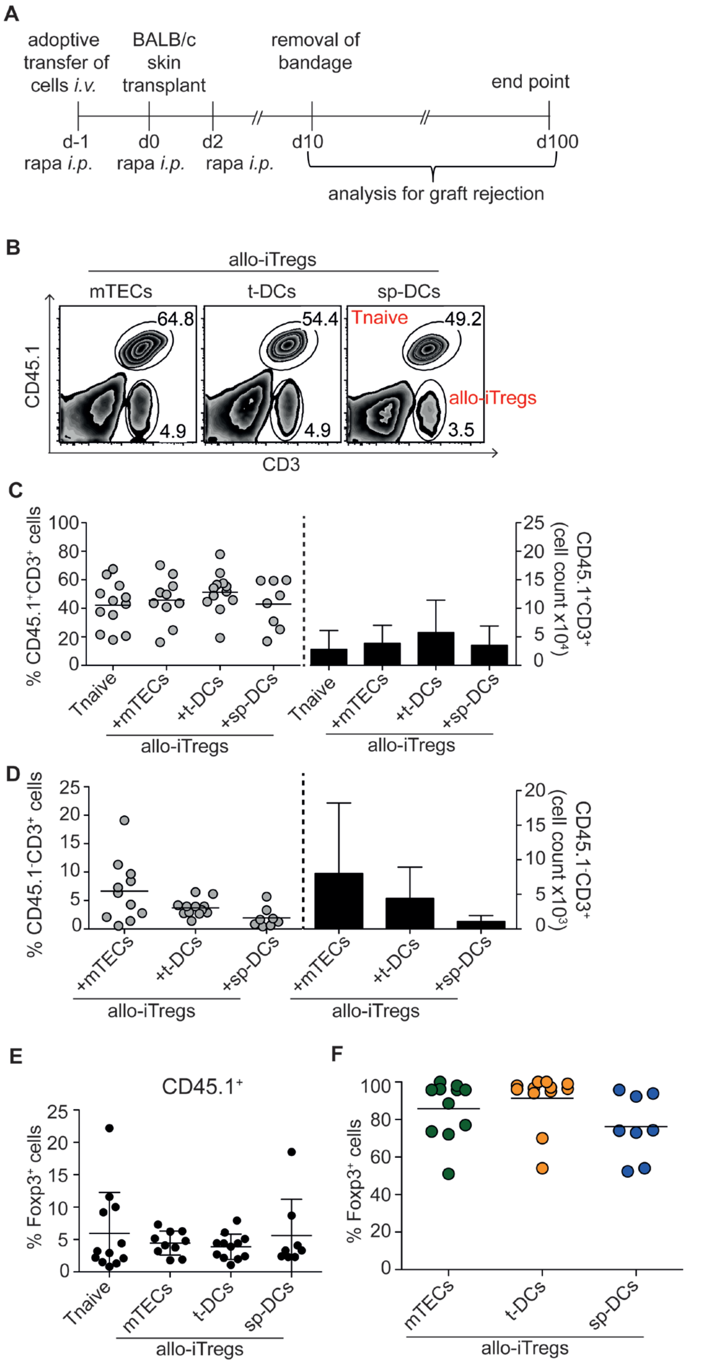

Supplementary Figure S8

Supplementary Figure S8: *In vivo* analysis of frequency and absolute numbers of T cells in lymph nodes draining the skin transplantation site. (A) Experimental set up for allogeneic skin transplantation. (B-F) Upon graft rejection or at day 100, the lymph nodes draining the skin transplantation site were analyzed for CD45.1, CD4, CD3 and Foxp3 expression by flow cytometry. (B) Representative zebra plots show $CD45.1^{+}CD3^{+}$ rejection-inducing naïve T cells and $CD45.1^{-}CD3^{+}$ allo-iTregs. Numbers indicate frequency of cells within corresponding gates. (C) Graph shows frequency (left) and absolute number (right) of progeny from $CD45.1^{+}CD3^{+}$ rejection-inducing naïve T cells within living cells from indicated groups. (D) Graph shows frequency (left) and absolute number (right) of progeny from $CD45.1^{-}CD3^{+}$ allo-iTregs within living cells from indicated groups. (E) Graph shows frequency of *de novo* induced $Foxp3^{+}$ cells among $CD45.1^{+}CD3^{+}$ T cells from indicated groups. (F) Graph shows stability of Foxp3 expression in $CD45.1^{-}CD3^{+}$ allo-iTregs derived from indicated groups. (B-F) Data are cumulative of three independent experiments. Naïve T cells only (n=12), mTEC allo-iTregs (n=11), t-DC allo-iTregs (n=12), sp-DC allo-iTregs (n=8). Symbols represent values from individual mice, and lines indicate mean. Bar graphs show mean ± SD.
