# Supplementary material for: Unique properties of thymic antigen-presenting cells promote epigenetic imprinting of alloantigen-specific regulatory T cells
Source: Oncotarget. 2017 Mar 15;8(22):35542–57. doi: 10.18632/oncotarget.16221 (PMC5482597; doi:10.18632/oncotarget.16221)
Supplement: Supplementary file 5 [file oncotarget-08-35542-s005.docx]

**Supplementary Table 4. Mapping statistics from *Tophat2* read alignment versus the mouse genome.** RNA‑Seq was performed on triplicate samples from indicated APCs.

|  | **mTECs** | | | **sp‑DCs** | | | **t‑DCs** | | |
| --- | --- | --- | --- | --- | --- | --- | --- | --- | --- |
|  | M1 | M2 | M3 | S1 | S2 | S3 | T1 | T2 | T3 |
| **#Reads**  **M (million)** | 28.5 M | 27.4 M | 31.2 M | 44.2 M | 35.3 M | 44.2 M | 31.6 M | 72.5 M | 31.4 M |
| **#Mapped**  **M (million)** | 26.1 M | 25.1 M | 28.9 M | 40.4 M | 32.2 M | 38.0 M | 29.2 M | 63.7 M | 29.0 M |
| **Mapping rate** | 91.5 % | 91.4 % | 92.6 % | 91.3 % | 91.1 % | 85.9 % | 92.4 % | 87.8 % | 92.5 % |
