# Supplementary material for: Unique properties of thymic antigen-presenting cells promote epigenetic imprinting of alloantigen-specific regulatory T cells
Source: Oncotarget. 2017 Mar 15;8(22):35542–57. doi: 10.18632/oncotarget.16221 (PMC5482597; doi:10.18632/oncotarget.16221)
Supplement: Supplementary file 6 [file oncotarget-08-35542-s006.docx]

**Supplementary Table 5. Primers for TSDR pyrosequencing.** Targeted gene locus, primer name, orientation and primer sequences are depicted.

| **Gene** | **Name** | **Direction** | **Sequence** |
| --- | --- | --- | --- |
| Foxp3 | mTSDR-bPyro-5 | Forward | biotin-AAGGGGGTTTTAATATTTATGAGG |
|  | mTSDR-Pyro-8 | Reverse | AAACCCTATTATCACAACCTAAACTTA |
|  | mTSDR-Seq12-9 | Sequencing | AACCAAATTTTTCTACCATTA |
|  | mTSDR-Seq8-6 | Sequencing | ACAAATAATCTACCCCAC |
|  | mTSDR-Seq5-4 | Sequencing | AATAAACCCAAATAAAATAATATAAAT |
